# Identifying concerted evolution and gene conversion in mammalian gene pairs lasting over 100 million years

**DOI:** 10.1186/1471-2148-9-156

**Published:** 2009-07-07

**Authors:** Andrew R Carson, Stephen W Scherer

**Affiliations:** 1 The Centre for Applied Genomics, Program in Genetics & Genome Biology, The Hospital for Sick Children, Toronto, Ontario, Canada; 2 Department of Molecular Genetics, University of Toronto, Ontario, Canada

## Abstract

**Background:**

Concerted evolution occurs in multigene families and is characterized by stretches of homogeneity and higher sequence similarity between paralogues than between orthologues. Here we identify human gene pairs that have undergone concerted evolution, caused by ongoing gene conversion, since at least the human-mouse divergence. Our strategy involved the identification of duplicated genes with greater similarity within a species than between species. These genes were required to be present in multiple mammalian genomes, suggesting duplication early in mammalian divergence. To eliminate genes that have been conserved due to strong purifying selection, our analysis also required at least one intron to have retained high sequence similarity between paralogues.

**Results:**

We identified three human gene pairs undergoing concerted evolution (*BMP8A/B*, *DDX19A/B*, and *TUBG1/2*). Phylogenetic investigations reveal that in each case the duplication appears to have occurred prior to eutherian mammalian radiation, with exactly two paralogues present in all examined species. This indicates that all three gene duplication events were established over 100 million years ago.

**Conclusion:**

The extended duration of concerted evolution in multiple distant lineages suggests that there has been prolonged homogenization of specific segments within these gene pairs. Although we speculate that selection for homogenization could have been utilized in order to maintain crucial homo- or hetero- binding domains, it remains unclear why gene conversion has persisted for such extended periods of time. Through these analyses, our results demonstrate additional examples of a process that plays a definite, although unspecified, role in molecular evolution.

## Background

Over the past few decades, gene duplications have been recognized as one of the main forces capable of generating large gene families with novel functions. In fact, duplications are thought to be one of the primary sources of adaptive evolution given that they generate new genetic material, and in doing so, create substrates that can undergo divergence through mutations [1]. Thus, gene duplications have the potential to affect genomic evolution dependent on the results of these mutations.

Duplicated genes are subject to the same forces that affect the evolution of single copy genes. These include genetic drift, which is opposed by negative or purifying selection, and positive selection, which can fix advantageous changes faster than expected by chance [2-4]. Additionally, other forces affect the evolution of duplicated genes. Functional redundancy can permit the accumulation of changes in one copy of the gene without negative consequences to an organism's proteome. Although this often leads to silencing or deletion of one gene copy, it can also lead to improvements on the ancestral functions or the development of new functions [5-9]. Subfunctionalization [6,7], where an ancestral gene's functions are shared between the descendant genes, and neofunctionalization [5], where one copy acquires a novel gene function, are possible consequences of divergence following gene duplication.

Most duplicated genes tend to diverge over time [1,10]. However, in some instances the genes, or parts of the genes, evolve together in a process known as concerted evolution [8,9,11]. Essentially, instead of gene sequences becoming progressively more dissimilar, the sequences remain highly similar or even identical. Although low divergence can also be explained by strong purifying selection, these two phenomena can be distinguished by comparisons across species. The hallmark of concerted evolution is that high sequence similarity between genes is maintained within a species (between paralogues) while divergence occurs between species (between orthologues). This is distinct from strong purifying selection, where divergence is impeded both within and between species such that the function, which is highly susceptible to changes, is preserved.

Concerted evolution between duplicated genes can be caused by ongoing genetic exchange called gene conversion [12-14]. Gene conversion is the non-reciprocal exchange of genetic material between homologous sequences. This process can have both positive and negative consequences. Beneficially, gene conversion can decrease mutational load, eliminate deleterious mutations, and spread advantageous alleles, thus playing a role in adaptive evolution [13,14]. Conversely, gene conversion can produce harmful phenotypes, such as Gaucher disease [15] and Shwachman-Diamond syndrome [16], when disruptive mutations from a pseudogene are substituted into its functional duplicate. The duration and frequency of the exchange between duplicated sequences is thought to be variable [11] and appears to depend upon several factors, including the distance between sequences. Several studies have shown a negative correlation between the frequency of gene conversion and the distance between homologous sequences [17,18], with a drop-off in frequency at distances greater than 55 kb [19]. Additionally, regions undergoing gene conversion may be disrupted by a few key mutations, such as the insertion of mobile elements or by the gradual accumulation of single nucleotide mutations [20]. Gene conversion can be a stochastic process, and it is unclear what effect selection has on the duration of gene conversion.

Gene conversion is also variable in the amount of gene sequence involved. While some entire gene sequences have undergone concerted evolution, others have a mosaic evolution pattern. Under mosaic evolution, segments of the gene are homogenized and evolve in concert, while others diverge without gene conversion [21]. This can complicate phylogenetic reconstruction, often producing trees that appear to indicate that multiple, independent and parallel duplications have occurred [20,22].

Characteristically, regions undergoing gene conversion have an elevated GC content relative to flanking sequences [23-25]. Intragenically, this can be seen within introns as well as at the third codon positions within exons (GC3). An increased GC content can be explained by the biased gene conversion (BGC) model, which asserts that heteroduplexes formed during gene conversion are preferentially repaired to GC alleles over AT alleles [24,25]. This leads to an increase in GC fixation and an elevated GC content. Biased DNA repair has been reported in mammalian cells, lending weight to this model [26]. This characteristic increase in GC content can be used in support of other evidence that a region is undergoing gene conversion.

Here we performed a whole genome analysis to look for duplicated genes undergoing concerted evolution. Previous whole genome analyses have been conducted looking for gene conversion in *C. elegans *[17], *S. cerevisiae *[18], rodent genomes [19], bacterial genomes [27], the rice genome [28] and *D. melanogaster *[29]. Until recently [30], there were no whole genome analyses of gene conversion in the human genome. Benovoy and Drouin [30] recently used a whole genome approach to identify examples of gene conversion in the human genome. However, their analysis was restricted to multigene families with three or more members. Our study differs in that we attempt to identify gene pairs (gene families with only two members) created by duplication early in mammalian radiation that have maintained regions of high sequence similarity due to ongoing gene conversion.

Our goal was to identify examples of gene conversion preserved in all, or a large majority, of mammalian species. A small number of other genes, such as *Oxct2a *and *Oxct2b*, *EMR2 *with *CD97 *and *EMR3*, *TLR1 *and *TLR6*, and *CCR2 *and *CCR5 *[31-34], have been reported to be evolving in concert by gene conversion in multiple lineages. By looking for duplicated genes in both the human and mouse genomes with greater sequence percent identity between paralogues than between orthologues, we identified three gene pairs with signals of long enduring concerted evolution: *BMP8A/B*, *DDX19A/B*, and *TUBG1/2*. Two of these (*DDX19A/B*, and *TUBG1/*2) are novel examples, while one (*BMP8A/B*) has been reported previously [34]. We also detail a detection strategy that can be applied to identify additional examples of genes evolving by this phenomenon. We performed extensive phylogenetic studies and analyzed the selective pressures acting on the gene pairs as well as their relative expression patterns in several human tissues. Through these examinations we show that gene conversion has been occurring between the gene pairs for over 100 million years, and the extended duration of this process in multiple distant lineages suggests that there has been prolonged selection for homogenization of segments within the genes.

## Results

### Identification of Gene Pairs

**Figure 1:**
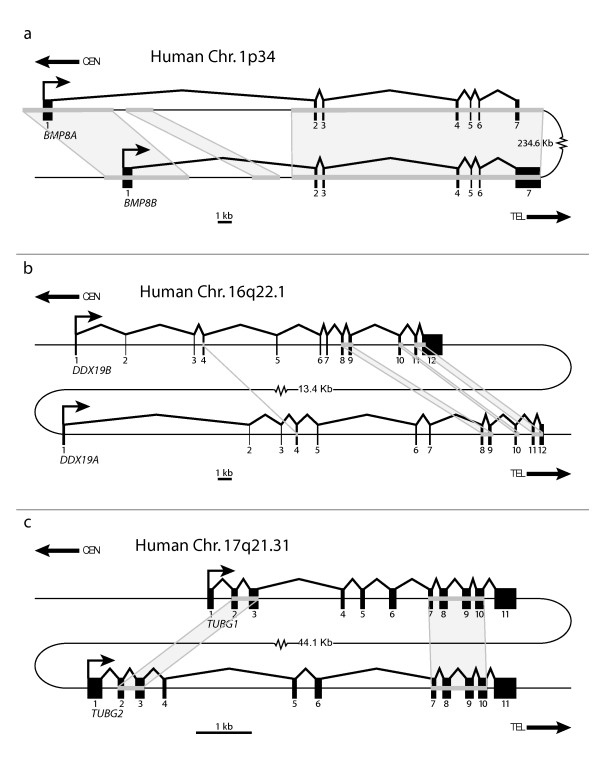
**Structure and orientation of gene pairs**. Gene structures of a) *BMP8A/B*, b) *DDX19A/B *and c) *TUBG1/2 *are presented. While *BMP8A/B *are in an inverted orientation with respect to each other, *DDX19A/B *and *TUBG1/2 *are in a direct orientation. The chromosomal band and distances between the gene pairs is also shown. Light grey boxes represent regions that show high sequence identity (>97%) between the gene pairs (determined by mVISTA).

Using our search criteria, outlined in Methods [also see Additional file 1], we were able to identify three gene pairs as candidate loci for concerted evolution. Due to the filtering steps we utilized, each gene pair is less than 1 Mb apart and shares high sequence similarity (>97%) in at least two consecutive exons and their intervening intron. The three gene pairs we identified are: *BMP8A/B*, *DDX19A/B*, and *TUBG1/2*. Figure 1 shows the gene structure and orientation of these gene pairs in the human genome.

*BMP8A *and *BMP8B *are members of the bone morphogenesis protein family, a subfamily of the transforming growth factor type beta (TGF-β) supergene family, found on chromosome 1p34 approximately 235 Kb apart in an inverted orientation. Each gene has seven exons with an open reading frame (ORF) of 1209 bps encoding a protein containing 402 amino acids. Bone morphogenesis proteins are known to be involved in vertebrate development [35,36]. While little has been reported about their function in humans, this gene pair has been linked to involvement in reproductive system development in the mouse (*Bmp8a *is expressed in the epididymis in males and the decidual cells of the uterus in pregnant females; *Bmp8b *is expressed in the germ cells of the testis in males and the trophoblast cells of the placenta in pregnant females) [37,38]. Concerted evolution of this gene pair was previously reported [34].

*DDX19A *and *DDX19B *are DEAD box helicase genes that are found on chromosome 16q22.1. These genes are tandemly duplicated (direct orientation) 13 Kb apart. Both genes have 12 exons but differ by 3 bps (CTG) in their ORFs. While *DDX19A*'s ORF transcript size is 1437 bps (encoding a 478 aa protein), *DDX19B *has an additional codon (1440 bps ORF encoding a 479 aa protein). Using chicken, which has a single orthologue of *DDX19*, as an outgroup, it is apparent that *DDX19A *has undergone a deletion of 3 bps near the start of exon 3. This deletion is seen in all investigated mammalian orthologues of *DDX19A*, including opossum. Although the functions of these genes are not well characterized, both genes are thought to be ATP-dependent RNA helicases that are involved in mRNA transport from the nucleus [39].

*TUBG1 *and *TUBG2 *are gamma tubulin genes on chromosome 17q21.31 and are situated in direct orientation approximately 45 Kb apart. Both genes have 11 exons with an ORF size of 1356 bps, encoding a protein with 451 amino acids. The gamma tubulin genes are components of the microtubule organizing centers where they play a central role in the nucleation of microtubules [40,41].

### Identification of Orthologues

Using various techniques, including BLAT [42], BLAST [43] and RT-PCR, we identified homologues of each gene in additional organisms. In all three cases, orthologues of both copies within the gene pair were identified in eutherian mammals, while only a single orthologue was found in non-mammalian vertebrates. Also, in each mammalian species investigated, only two copies of each gene could be identified, indicating that these multigene families have a consistent copy number of two in mammals. Interestingly, only a single orthologue of *BMP8A/B *was found in the opossum, while orthologues appear to exist for both genes from the *DDX19A/B *and *TUBG1/2 *gene pairs in this organism. Although the low sequence coverage and incomplete assembly of the opossum genome could prevent the identification of a second *BMP8 *orthologue, another explanation involves different timings for the origins of these gene pairs. In each case, since two orthologues are present in all eutherian mammals examined, the most parsimonious explanation is that a duplication event occurred early in mammalian evolution. For *BMP8A/B*, the absence of the duplication in opossum suggests that the duplication event occurred after the divergence of the opossum lineage (order *Marsupialia*) from the armadillo (order *Edentata*). This would place the duplication event somewhere between 129 and 173 million years ago (MYA) [44]. For *DDX19A/B *and *TUBG1/2*, the presence of the duplication in opossum suggests that the duplication event is older, occurring before the divergence of opossum lineage (>173 MYA).

### Phylogenetic Analyses

**Figure 2:**
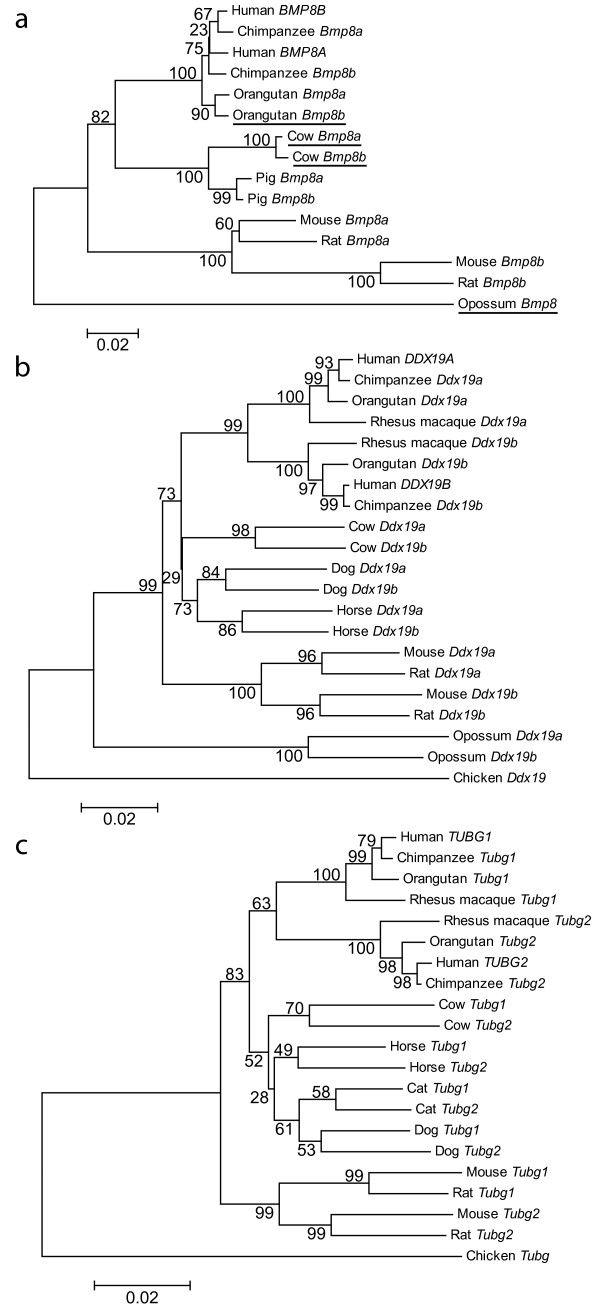
**Neighbor-joining trees of gene pairs**. Neighbor-joining trees of gene pairs a) *BMP8A/B*, b) *DDX19A/B *and c) *TUBG1/2 *were created in MEGA3 using a Tamura-Nei model of sequence evolution. Opossum *Bmp8*, chicken *Ddx19 *and chicken *Tubg *were used as outgroups to root the trees. Bootstrap values (10,000 replicates) are shown on the interior branches of the tree. A distance scale is shown below each tree. Underlined species in a) are missing exon 1, preventing the use of this exon in the phylogenetic analysis (see Methods). Additionally, maximum likelihood trees were created for comparison and show highly similar patterns of evolution with the above trees [see Additional file 2].

We created phylogenetic trees using two different methods, Neighbor-joining (Figure 2) and maximum likelihood [see Additional file 2], to examine the evolution of the duplicated gene pairs in the mammalian lineage. Comparison of the trees created by these two different methods showed that the branching pattern and node structure were highly similar in every case.

Generally, duplicated genes that undergo divergent evolution display a tree in which the orthologues cluster and are monophyletic. In contrast, duplicated genes that evolve in a concerted fashion display trees where paralogues cluster. Examining the phylogenetic trees in Figure 2, it is apparent that these trees show signatures of both types of evolution. For example, in Figure 2b, *DDX19A *and its three primate orthologues are monophyletic, as is *DDX19B *and its primate orthologues. A similar phylogeny is also seen in the rodent lineage. This pattern is typical of divergent evolution. In contrast, the pattern for dog, cow, horse and opossum is distinct in that the paralogues group together. This could be indicative of concerted evolution. An alternate explanation for this pattern involves multiple independent duplications. *DDX19A/B*'s phylogeny could be explained by six independent duplications (in primates, rodents, dog, cow, horse and opossum). However, a single duplication that predates the mammalian divergence would be a more parsimonious explanation. In this hypothesis, mosaic evolution, where some exons evolve divergently and others evolve concertedly, would explain the mixture of evolutionary patterns seen in the phylogenetic trees.

**Figure 3:**
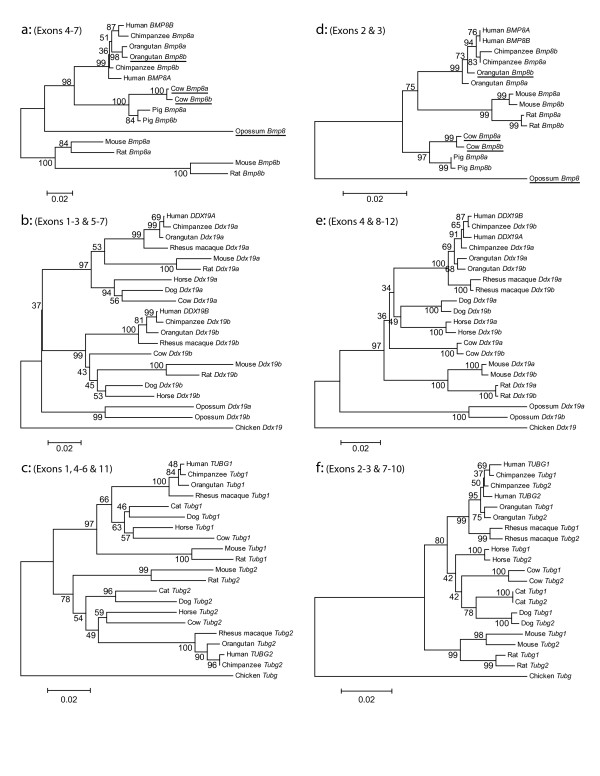
**Neighbor-joining trees showing mosaic evolution within gene pairs**. Neighbor-joining trees of gene pairs a) and d) *BMP8A/B*, b) and e) *DDX19A/B *and c) and f) *TUBG1/2 *were created in MEGA3 using a Tamura-Nei model of sequence evolution. Exons were divided into two categories and phylogenetic trees, showing a), b) and c) divergent evolution or d), e) and f) concerted evolution, are illustrated. Opossum *Bmp8*, chicken *Ddx19 *and chicken *Tubg *were used as outgroups to root the trees. Bootstrap values (10,000 replicates) are shown on the interior branches of the tree. A distance scale is shown below each tree. Underlined species in a) and d) are missing exon 1, preventing the use of this exon in the phylogenetic analysis. Additionally, maximum likelihood trees were created for comparison, showing highly similar patterns of evolution with the above trees [see Additional file 5].

To test this hypothesis, we performed a sliding window analysis on the gene pairs [see Additional files 3 and 4] then built new phylogenetic trees (Figure 3) after separating the exons into divergently or concertedly evolving categories [also see Additional file 5]. Both analyses favor the conclusion that these gene pairs are evolving in a mosaic pattern. The exons were categorized as divergently or concertedly evolving using a combination of the mVISTA identities shown in Figure 1, the sliding window plots [see Additional files 3 and 4], and a visual inspection of the multiple alignments. Figure 3 shows that categorizing the exons before building phylogenetic trees is sufficient to separate the two signatures of evolution observed in Figure 2 [also see Additional file 5]. The divergent gene trees for *DDX19A/B *and *TUBG1/2 *(Figure 3b and 3c) show monophyletic clades in which all the eutherian mammal orthologues cluster. This phylogeny is not seen for *BMP8A/B*, except for in the rodent lineage, but can be explained by the greater stretch of high similarity between paralogues in non-rodent mammals (Figure 1 and sliding window analysis [see Additional files 3 and 4]). In contrast, the convergent gene trees for all three gene pairs (Figure 3d, e and 3f) have paralogues grouping together in almost all instances. The only exceptions are the human and chimpanzee branches in *DDX19A/B *and *TUBG1/2*. These phylogenetic trees illustrate that these gene pairs have evolved in a mosaic pattern. A summary of the percent similarity between the human genes in divergent and concerted regions was also generated [see Additional file 6].

### Evidence of Gene Conversion

One cause of concerted evolution is gene conversion. To look for statistically significant evidence of homogenization, we used the program Geneconv [45]. Multiple alignments, both of the ORFs and the genomic sequences, were used as input for Geneconv in order to detect candidate fragments of aligned gene conversion. The results identify several statistically significant fragments in both the ORF alignments and the genomic sequence alignments (Table 1). From the table, it is clear that the regions detected by Geneconv overlap to a large degree with the peaks of high sequence identity between paralogues (Figure 1 and the sliding window analysis [see Additional files 3 and 4]).

**Table 1:** Positive fragments identified by Geneconv

	ORF^1^		Genomic DNA^1,2^	
	
	Primate	Rodent	Human	Mouse
*BMP8A/B*	(Exons 1–5^h^)	(Exons 2–4^r^)	Exon 1-Intron 1	Intron 1-Intron 3
			Intron 3-Intron 6	Intron 4-Intron 5

*DDX19A/B*	Exons 8–12^a^	Exons 8–12^a^	Exon 8-Intron 8	Exon 8-Exon 12
			Exon 10-Intron 10	
			Intron 10-Intron 11	

*TUBG1/2*	Exons 2–3^h, c, rh^	Exons 6–10^a^	Intron 1-Intron 3	Intron 2-Exon 3
	Exons 7–11^c, o, rh^		Intron 6-Intron 7	Exon 7-Intron 10
			Intron 8-Intron 10	

^1 ^Exons were included if >50 bps were within the positive fragment.

^2 ^Introns were included if >100 bps were within the positive fragment.

^h ^Human, ^c ^chimp, ^o ^orangutan, ^rh ^rhesus macaque, ^r ^rat, ^a ^all primates or rodents.

Hits enclosed by brackets are positive pairwise fragments only. P-value for that region in the global analysis is not significant (>0.05).

Hits within genomic DNA span both introns and exons.

**Figure 4:**
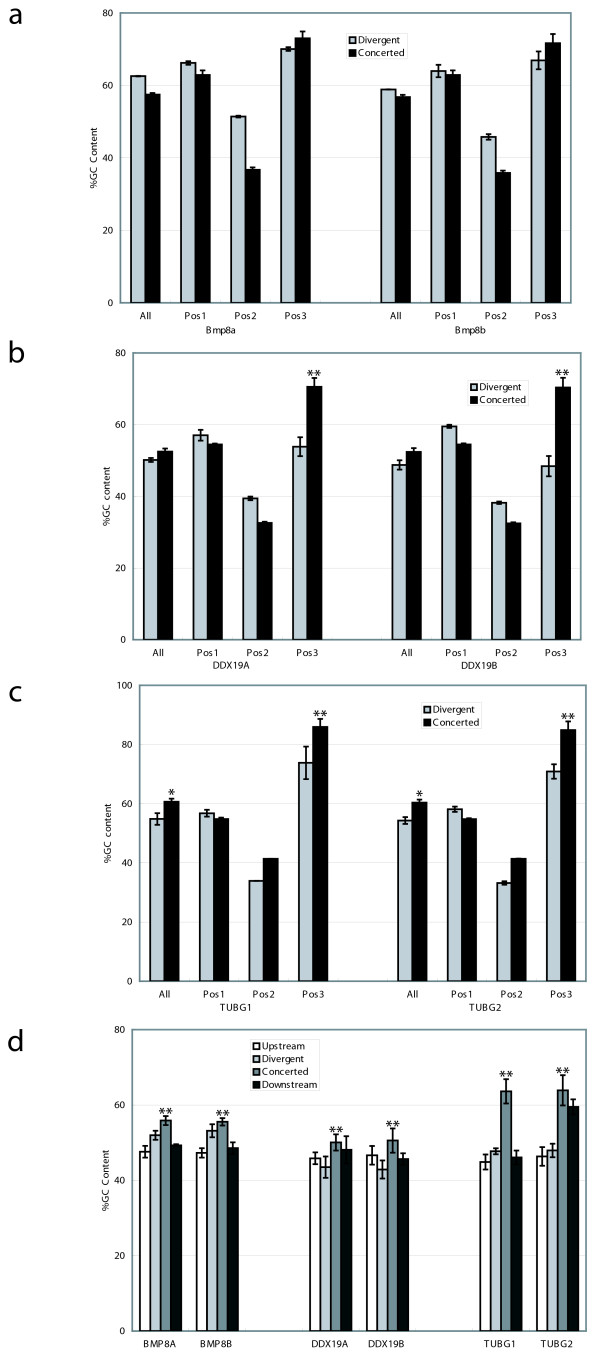
**GC content analysis within the gene pairs**. Charts compare the GC content of regions evolving in concert with regions evolving divergently. The GC contents at each codon position within gene pairs a) *BMP8A/B*, b) *DDX19A/B *and c) *TUBG1/2 *are shown, along with d) the GC content of introns and 10 kb of upstream and downstream flanking sequence of each gene. GC contents were calculated as an average of five (*BMP8A/B*) or eight (*DDX19A/B *and *TUBG1/2*) multiple sequences (available upon request). Statistical tests (Fisher-Exact test and Yates-corrected Chi-square test) were calculated using the GC content in the human sequences and used to look for significance. P-values less than 0.05 are indicated by *, while P-values less than 0.01 are indicated by **.

Increased GC content at the third codon position and in introns has also been used as evidence for gene conversion [25,33]. GC content was calculated for each gene in both the divergent and concerted regions in multiple species. The average GC content was calculated at each of the codon positions and plotted with p-values ascertained within the human sequences using a Fisher Exact Test and/or Yates-corrected Chi-square test (Figure 4). For *BMP8A/B*, only rodent sequences were used because in other species all of the exons appear to be evolving in concert. From these plots, it is clear that there is a significant difference (P < 0.05) in the third codon position for *DDX19A/B *and *TUBG1/2 *(Figure 4b, c). However, although there is an increase in the third codon position of the rodent *Bmp8a/b *(Figure 4a), the difference is not significant. A similar comparison was undertaken using the introns from these three gene pairs. Again, a significant increase in GC content is seen in the introns that appear to be evolving under concerted evolution, as opposed to the introns evolving divergently as well as 10 kb of upstream and downstream flanking sequence (Figure 4d). Taken together with the results from Geneconv, the increase in GC content suggests that gene conversion is the cause of the concerted evolution in these three gene pairs.

### Sequence Evolution and Functional Domains

We investigated the selective pressures working on these gene pairs by calculating the dN/dS ratios between the orthologues. Of interest was whether the regions evolving divergently were under different selective pressures than the regions evolving in concert. Average dN/dS values, calculated using pairs of orthologues, are shown in Table 2. Values of dN/dS are calculated for the whole gene, as well as the regions evolving divergently and concertedly (as previously defined). As you can see from this table, five of the six values of dN/dS for the concerted regions are lower than the dN/dS values for the divergent regions. This suggests that either these regions are under more negative selective pressure or it could indicate that a consequence of gene conversion is lower nonsynonymous divergence between sequences.

**Table 2:** Comparison of dN/dS values of divergently and concertedly evolving regions

	Average dN/dS		
	
Gene Pair	Whole Gene	Divergent Region(s)	Concerted Region(s)
*BMP8A*	0.2290	0.1654	0.3879

*BMP8B*	0.2868	0.3016	0.2576

*DDX19A*	0.0384	0.0486	0.0305

*DDX19B*	0.0365	0.0486	0.0286

*TUBG1*	0.1210	0.2059	0.0741

*TUBG2*	0.1214	0.1780	0.0803

We also performed sliding window and PAML analyses to further examine the dN/dS values in the gene pairs [see Additional files 7 and 8]. Although the results from some analyses show suggestions of positive selection, no significant evidence was found. Therefore, there does not appear to be an overlap between the regions undergoing concerted evolution and positive selection. Conversely, there is some evidence indicating that the regions undergoing concerted evolution have lower dN/dS values, suggesting that both gene conversion and purifying selection are acting at these sites.

Additionally, we looked to see if the regions undergoing concerted evolution contained or overlapped with specific functional domains within these genes. However, in each case there did not appear to be any significant overlap of the functional domains with the regions undergoing concerted evolution [see Additional file 9]. Although parts of some domains overlap regions of concerted evolution, there is no clear relationship that could indicate the selection for gene conversion limited to a particular domain. In some cases, the exons involved in concerted evolution fall outside of the known functional domains. This could indicate that there is another functional domain in these exons that has not been previously described.

### Gene Expression Analysis

We performed gene expression analyses to determine whether there is differential tissue expression within the gene pairs. We used qPCR and pyrosequencing assays to attain a ratio of expression between the two members of the gene pair. However, the *BMP8A/B *gene pair was resistant to both assays. Therefore, although we were able to obtain measurements of expression for *DDX19A/B *and *TUBG1/2*, the expression analysis of *BMP8A/B *is limited to previously published reports in mice.

**Figure 5:**
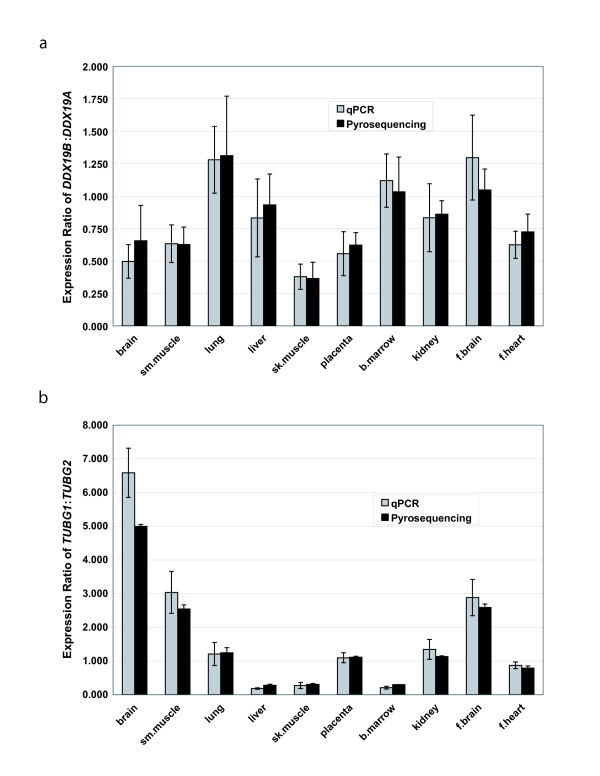
**Relative expression level comparisons with the gene pairs**. Charts show the ratio of expression between a) *DDX19A/B *and b) *TUBG1/2 *in ten tissues. Expression values were obtained using both qPCR (grey bars) and pyrosequencing (black bars). An expression ratio of 1.0 would indicate equal expression.

For *DDX19A/B*, we were able to use both qPCR (exon 7) and pyrosequencing (exons 8 and 10) to obtain expression ratios (Figure 5a). Although the values for each assay are slightly different, the overall trend is consistent. There appears to be several tissues where the expression level of the two genes is similar (lung, liver, bone marrow, kidney and fetal brain). However, in several other tissues the expression level of *DDX19A *is higher than the expression level of *DDX19B *(brain, smooth muscle, skeletal muscle, placenta, and fetal heart). In these tissues, it appears that *DDX19B *is expressed at about one half to three quarters the level of *DDX19A*, with the highest degree of differential expression seen in skeletal muscle.

Although the expression level difference between *DDX19A *and *DDX19B *is not considerable in most tissues, the expression ratio for *TUBG1 *and *TUBG2 *is more remarkable (Figure 5b). Again we used both qPCR (exon 11) and pyrosequencing (exon 8) to obtain expression ratios. Similarly, both assays are relatively consistent in their findings, although there are some small differences in their absolute values. Interestingly, for this gene pair there are several tissues where there is a significant difference in the level of expression between the two genes. Unlike *DDX19A/B*, where either the expression was equal or one gene (*DDX19A*) was higher in expression, there are some tissues where *TUBG1 *shows higher expression (liver, skeletal muscle and bone marrow), some tissues where *TUBG2 *shows higher expression (brain, smooth muscle and fetal brain) and some tissues where expression is relatively equal (lung, placenta, kidney and fetal heart). Also, the magnitudes of the expression differences are considerably larger in this gene pair, ranging from four to five times higher expression of *TUBG1 *in liver and bone marrow, to five to six times higher expression of *TUBG2 *in brain. Thus, the *TUBG1/2 *gene pair is differentially expressed in several tissues.

We performed additional analysis looking for regulatory regions upstream of each gene to try to assess whether gene pairs share promoters or enhancers. However, in general it appears that the upstream regulatory regions have diversified throughout the gene pair evolution, even though regions within the genes have evolved in concert [see Additional file 10].

## Discussion

We have identified three duplicated gene pairs arising early in mammalian evolution that have undergone concerted evolution via gene conversion. While one of these gene pairs has been previously reported to be evolving in concert [34], the other two pairs are novel. These findings, identified using a genome-wide strategy, increase by 50% the number of identified cases of human gene pairs undergoing this process for such an extended period of time. In each case, the two copies of the gene are separated by less than 250 kb of sequence in the human genome and we have inferred that duplication occurred over 129 MYA.

Phylogenetic analyses demonstrate that each pair of genes has regions evolving in concert in all examined mammalian species (Figure 3). Concerted evolution in multigene families is thought to occur by two mechanisms: unequal crossing over and gene conversion. While unequal crossing over is often invoked to explain concerted evolution within tandemly-arrayed gene families, gene conversion is used to explain concerted evolution of duplicated genes (copy number of two) and mosaic evolution [9,20,21]. This is because gene conversion can homogenize parts of a gene without changing the copy number. Since all of the eutherian mammals examined have evidence of only two copies of these genes, it is unlikely that unequal crossing over is the basis for the observed concerted evolution. For additional proof of gene conversion, we used the program Geneconv and also looked for a significant increase in GC content. Although homogenization or increased GC by themselves do not prove gene conversion is acting within these regions, in combination with mosaic evolution (Figures 1 and 3) and a consistent copy number (two genes), this evidence supports gene conversion, rather than unequal crossing over, as the cause of the concerted evolution in these gene pairs. Also, although the homogenization of the gene pairs could have been achieved by a single gene conversion event in each mammal, our evidence is more in line with multiple gene conversion events throughout evolution leading to concerted evolution of the gene pairs.

It is interesting that gene conversion between these gene pairs appears to have been active for over 100 million years and has resulted in concerted evolution in every mammal examined. The duration of gene conversion is thought to be variable and often stochastic in nature [11,20]. Therefore, although gene conversion could occur between two loci for over 100 million years by chance, it is expected to do so only in rare instances. Thus, it appears highly unlikely that gene conversion would be maintained coincidentally for over 100 million years in multiple lineages. Consequently, we speculate that if gene conversion is seen consistently at the same loci for an extended length of time, there may have been selection for this process and the maintenance of homogenized gene sequences.

The duration and prevalence of gene conversion within these gene pairs is suggestive of selection for homogenization and co-evolution. However, the basis for this proposed selection is unclear. Our analyses do not show any obvious overlap of the regions evolving in concert with protein domains or known functional sequences. Nevertheless, a few previous reports have identified similar patterns of concerted evolution which could provide clues as to why gene conversion can be maintained for such extended periods of time. Three of the reports describing similar gene pairs suggest that protein binding co-evolution between two genes might be the basis for the conserved gene conversion. The Vazquez-Salat *et al *[33] study, which looks at gene conversion between *CCR2 *and *CCR5*, suggests that along with homodimerization, these proteins also heterodimerize, creating a synergistic effect which enhances their function. Similarly, Kruithof [31] and Kwakkenbos [32] looked at gene conversion between *TLR1 *and *TLR6 *and between *EMR2*, *CD97 *and *EMR3*, respectively, and suggest that gene conversion may be used to conserve or co-evolve binding regions. These papers propose that mosaic evolution maintains structural or binding regions, while receptor, ligand binding or other functional domains are allowed to diverge. A similar proposal can be applied to explain the mosaic evolution identified in the three gene pairs we investigated. Additionally, the conserved boundaries of the mosaic evolution in multiple species further supports the idea that while some regions are co-evolving to maintain a specific function, other regions are diverging in both sequence and function.

Interestingly, if we look at the protein binding of the gene pairs we identified, there are some indications these proteins have conserved binding partners. We speculate that gene conversion has been utilized to maintain perfect homology at binding sites. Additionally, our results indicate that the regions undergoing gene conversion tend to have lower dN/dS values than the sequences evolving divergently (Table 2 and dN/dS sliding window analysis [see Additional files 7 and 8]). This suggests a second process, stronger negative selection in combination with gene conversion, has been utilized to conserve these sites. An example of binding site co-evolution occurs between *BMP8A *and *BMP8B*, which, like other BMP8 proteins, work as homodimers [46,47]. These proteins could theoretically interact and form heterodimers, similar to the interaction of BMPs -4 and -7 [48]. Perhaps a heterodimer would perform an additional or enhanced function. Similarly, by assessing TUBG1 and TUBG2 binding partners using NCBI's Entrez database [49] it is clear from *in vivo *evidence, based on affinity capture experiments, that both proteins interact with PXN, RNF19A, and TUBGCP3. In this case, although there is no evidence that the protein products of *TUBG1/2 *interact with each other directly, they may interact with the same partners and thus need to share a highly similar or identical binding domain. Although similar evidence is not available for *DDX19A/B*, shared binding partners may be identified by functional interaction analyses of these proteins. However, our assertion that gene conversion is being selected to maintain binding sites is purely speculative. While it appears that selection may be acting to maintain gene conversion within these regions, the purpose and evolutionary implications of this process are still largely unknown.

Our gene expression analyses of *DDX19A/B *and *TUBG1/2 *(Figure 5) indicate that although these genes could share some functions, they may act in different tissues. This is a form of subfunctionalization. An example occurs in rodent *Bmp8a/b*, which have been shown to have differential expression in the mouse reproductive system [37,38]. The most striking example of subfunctionalization was the differences seen in the expression between *TUBG1 *and *TUBG2*. These genes appear to have significantly different expression in six of the ten examined tissues (three with higher *TUBG1 *expression, and three with higher *TUBG2 *expression). These results agree with previously reported expression analyses of these genes, such as the increased expression of *TUBG1 *in liver and *TUBG2 *in brain [40,41]. This could indicate that the genes have different functional relevance in different tissues. We attribute the subfunctionalization of tissue expression patterns observed in the three gene pairs to the divergence of regulatory regions upstream of each gene. When we examine 10 kb of sequence upstream of each gene, we see little to no overlap in predicted or known enhancers and conserved transcription binding sites. These results again show the mosaic pattern of evolution within these gene pairs, where some regions evolve in concert to maintain function. Other regions, including regulatory regions, are not preserved, leading to diverging functions and subfunctionalization.

## Conclusion

In conclusion, it appears that gene or domain homogenization caused by extended periods of gene conversion can result in concerted evolution that may be maintained, perhaps by selection, throughout mammalian evolution. We speculate that this process is being used to maintain homo- or hetero- binding domains within gene pairs. We have identified additional examples of this relatively rare form of evolution and, although it is rare, postulate that it has relevance within gene duplication evolution

## Methods

### Identification of Gene Pairs

To search for gene pairs potentially undergoing concerted evolution, we initially used MEGABLAST [50] to compare the masked human genome (Build 36; hg18) against itself. We then filtered out self-hits to look for regions of the human genome that have high sequence identity to another region (>90% identity over 100 bp). Next, using the genomic coordinates of all Refseq genes, we look for duplicated regions that overlap genes. At this stage, these duplications could be intronic or exonic. The next step involved isolating genes that shared higher levels of sequence identity (>97%) spanning two consecutive exons and an intervening intron (exon x - intron x - exon x+1 matching exon y - intron y - exon y+1) [see Additional file 1]. At this stage, the dataset was large (containing 1260 hits) and comprised mainly of recently expanded gene families. To simplify our dataset, we next performed two filtering steps that greatly reduced the number of hits. We filtered for i) copy number, keeping only the hits that were present as a pair and ii) gene distance, keeping only the hits that were located less than 1 Mb apart. We filtered for copy number because this would eliminate large gene families that could be undergoing concerted evolution through the process of unequal crossing over. We filtered for gene distance since gene conversion seems to be enriched between sequences that are closer together [17-19]. The choice of a 1 Mb cutoff was arbitrary. However, it appears to be generous given that it has been shown that most examples of gene conversion have been found separated by less than 55 kb of intervening sequence [19].

The dataset (containing 182 hits) created by these filtering steps contained human duplicated gene pairs that share greater than 97% identity in at least two consecutive exons and the intervening intron and are less than 1 Mb apart. However, this dataset is predisposed to contain mostly recent duplications. To eliminate these, we manually compared our dataset with the mouse genome to see if a corresponding gene pair (or more copies) existed. If only a single gene was found in mouse, we concluded that the duplication was recent (occurred after the human-mouse divergence). If a gene pair was also seen in mouse, there are at least two possible explanations: the gene was independently duplicated in both the mouse and human genomes or the gene was duplicated a single time in a common ancestor (before human-mouse divergence). To rule out independent duplications, we manually looked for the duplication in other mammals (initially rat and dog, but then expanded to all other mammals available). In some instances the genes did appear to be independently duplicated (such as *ELA3A/B*, data not shown). However, an ancestral duplication was apparent in three of the gene pairs. These gene pairs were kept for further analysis.

### Analysis of Gene Pairs

Initial analysis of the gene pairs was performed using the UCSC genome browser [51]. Using the utilities and data present within this browser, we were able to obtain the sequence and structure of each gene of interest. We then used mVISTA [52,53] to compare the gene pairs' genomic sequences (exonic and intronic) with one another (Figure 1).

We then expanded our analysis to additional mammalian genomes. Using a combination of BLAT against the UCSC genome browser [42], BLAST against multiple NCBI databases including the trace archives [43] and RT-PCR using primers (available upon request) designed within highly conserved sequences of the gene, we were able to obtain sequences from multiple mammals. Due to the incomplete assembly of available DNA sequences from many organisms, we were occasionally unable to assemble the full-length sequence of one or more orthologues. In these instances, we eliminated the species from further phylogenetic analyses. However, for *BMP8A/B*, exon 1 has a high GC content which prevented PCR amplification of its sequence. Hence, we were unable to confirm this exon in four genes within the eight species with assembled sequences (4 of the 15 genes). Therefore, the phylogenetic analyses for this gene pair do not include exon 1.

Once these sequences were obtained, we used two different tools, MEGA3 [54] and PAUP [55] to analyze the phylogeny of the gene pairs. ClustalW [56] was used to align the cDNA sequences. This was followed by manual correction of the alignments. These corrected alignments were then used to create Neighbor-joining trees under the Tamura-Nei model of evolution in MEGA3 and replicated with 10,000 bootstraps. To verify the structure of these trees, we used PAUP [55,57] to create maximum likelihood trees. The likelihood settings used by PAUP were determined using MODELTEST [58] to select the best-fit model by hLRT (parameters available upon request). Using regions delimited by the mVISTA and sliding window [see Additional files 3 and 4] results, we performed additional phylogenetic analyses (same methods as described above) to compare the regions evolving divergently to the regions evolving in concert.

### Evidence of Gene Conversion

We used two methods to look for evidence of gene conversion. First we used Geneconv [45] to look for regions that have statistically significant evidence for gene conversion. For this program, we used alignments created by clustalW [56] (cDNA) and by CHAOS with DIALIGN [59] (genomic DNA) as the input. Geneconv was then able to detect candidate fragments of aligned gene conversion between the gene pairs (mismatches allowed).

Second, we looked for evidence of increased GC content in the third codon position and in introns of the regions undergoing concerted evolution. Using MEGA3, GC content was calculated in multiple sequences from both exonic and intronic sequences, as well as 10 kb of upstream and downstream flanking sequence of each gene. These sequences were divided into regions evolving in concert versus regions evolving divergently. GC contents were calculated as an average of five (*BMP8A/B*) or eight (*DDX19A/B *and *TUBG1/2*) multiple sequences. The GC content was compared between these regions and Fisher Exact and Yates-corrected Chi-square statistical tests were performed on the results to look for significant enrichment of GC in regions evolving in concert.

### Selection Analysis

We analyzed the selective pressures on the gene pairs by calculating the dN/dS ratio between orthologues. These ratios were calculated between pairs of orthologues using MEGA3. Values were calculated for the whole gene sequence, as well as divergently evolving and concertedly evolving subsections of the sequence. dN/dS values were averaged and then comparisons were drawn between the gene pairs and their different regions.

### Gene Expression Analysis

We used two techniques to look at the relative expression levels within the gene pairs. For the first, qPCR, we designed unique primers (available upon request) in diverged exons. These primers were chosen such that they did not span an intron-exon boundary and hence would amplify both cDNA and genomic DNA. Genomic DNA could then be used as a two copy control in the qPCR reaction. Thus, amplification of genomic DNA could be used to create a standard curve for both genes, normalizing the expression of each gene and allowing their ratio of expression to be calculated. Expression analysis was performed on 10 tissues (brain, smooth muscle, lung, liver, skeletal muscle, placenta, bone marrow, kidney, fetal brain and fetal heart).

For the second analysis, pyrosequencing, we designed primers in exons evolving in concert, in sequences that share 100% percent identity between the two paralogues. Thus, these primers would be used to amplify both copies within the gene pair. Each primer set contained a forward primer, a reverse biotinylated primer and a sequencing primer adjacent to a paralogous sequence variant (PSV) (sequences available upon request). These primers were designed within a single exon (such that both cDNA and genomic DNA would be amplified) following the PSQ 96 preparation guide. Pyrosequencing was performed using the Pyro Gold Enzyme Mixture (Biotage) and analyzed using PSQ 96MA 2.1 ID System and amplification of genomic DNA was used to normalize the expression levels (since PSV peaks in the genomic DNA should be in a 1:1 ratio). The pyrosequencing analyses were performed on the same 10 tissues as the qPCR analyses.

## Abbreviations

BGC: Biased gene conversion; ORF: Open reading frame; MYA: Million years ago; TFBS: Transcription factor binding sites; PSV: Paralogous sequence variant.

## Authors' contributions

While ARC was responsible for the acquisition, analysis and interpretation of data, both ARC and SWS were involved in the conception and design of the study and the preparation and revision of the manuscript. Both authors have read and approved the final manuscript.

## Supplementary Material

Additional file 1 **Flow chart of identification strategy**. This file shows a flow chart illustrating the method used to identify potential gene pairs that have undergone concerted evolution. Click here for file

Additional file 2 **Maximum likelihood trees of gene pairs**. This file shows maximum likelihood trees of gene pairs created in PAUP. Click here for file

Additional file 3 **Sliding window analysis calculating percent identity between orthologues and paralogues**. This file details a sliding window percent identity analysis that shows the mosaic evolution within the gene pairs. This figure shows the sliding window analysis plot with the percent identities calculated between orthologues and paralogues. Click here for file

Additional file 4 **Sliding window of percent identities figure**. This figure shows the sliding window analysis plot with the percent identities calculated between orthologues and paralogues Click here for file

Additional file 5 **Maximum likelihood trees showing mosaic evolution within gene pairs**. This file shows maximum likelihood maximum likelihood trees created in PAUP of gene pairs, in which the exons are divided into two categories showing divergent and concerted evolution. Click here for file

Additional file 6 **Description and percent similarity between gene pairs**. This file contains a table containing information about the gene pairs, including their ORF size, number of exons, and the percent identities of regions evolving divergently or concertedly. Click here for file

Additional file 7 **Sliding window analysis calculating dN/dS between paralogues and orthologues**. This file shows sliding windows used to calculate dN/dS between gene pairs. Click here for file

Additional file 8 **Sliding windows calculating dN/dS figure**. This figure shows the sliding window analysis plot with the dN/dS values calculated between gene pairs. Click here for file

Additional file 9 **Analysis of functional domains**. This file contains a description of the analysis looking for overlap between functional domains and regions evolving in concert. Click here for file

Additional file 10 **Analysis of upstream regulatory regions**. This file describes the analysis of predicted regulatory regions in the 10 kb of sequence upstream from each gene. It also contains a table showing the predicted regulatory regions in these sequences. Click here for file
