# Supplementary material for: Identifying concerted evolution and gene conversion in mammalian gene pairs lasting over 100 million years
Source: BMC Evol Biol. 2009 Jul 7;9:156. doi: 10.1186/1471-2148-9-156 (PMC2720389; doi:10.1186/1471-2148-9-156)
Supplement: Additional file 1 — Flow chart of identification strategy. This file shows a flow chart illustrating the method used to identify potential gene pairs that have undergone concerted evolution. [file 1471-2148-9-156-S1.pdf]

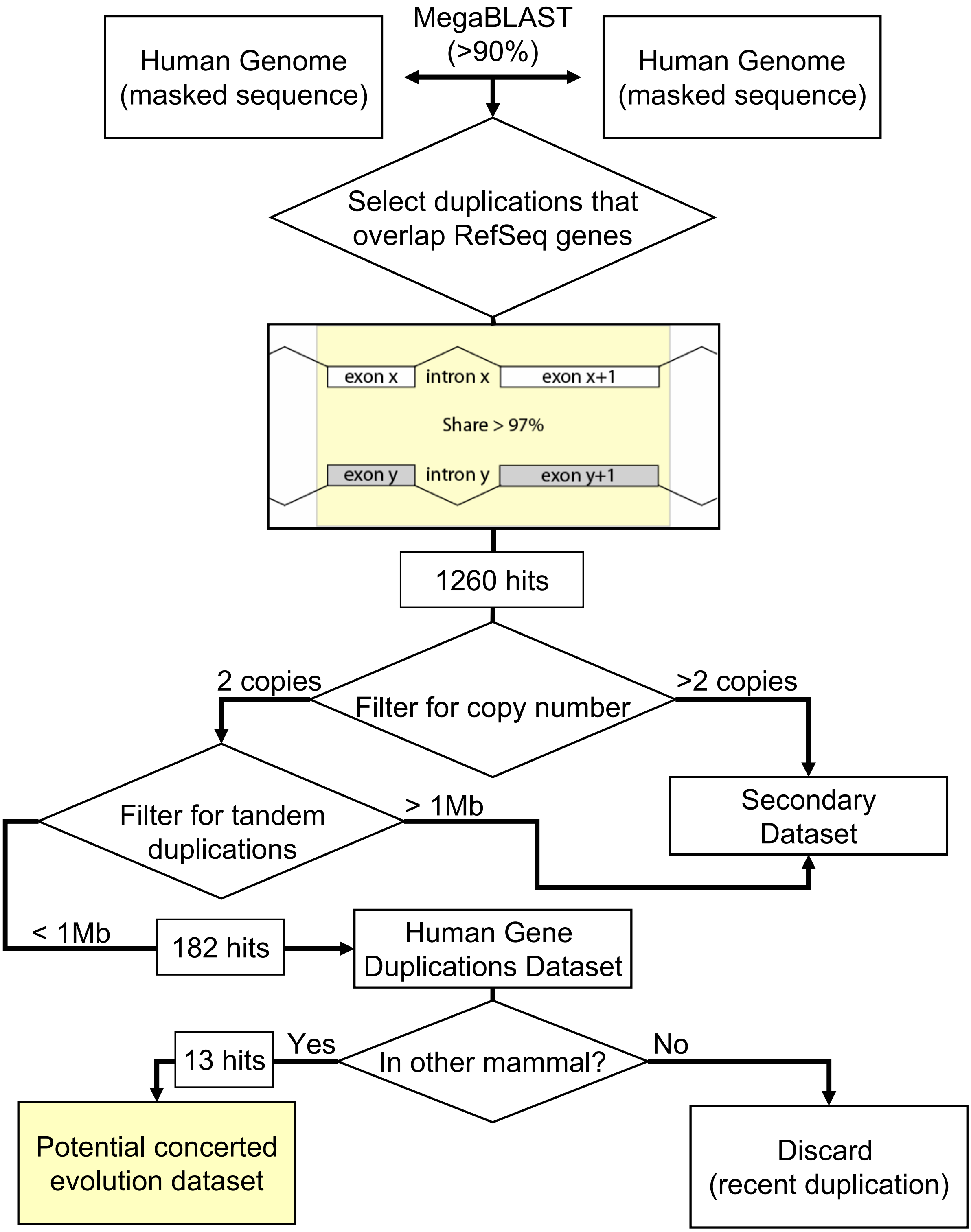

Additional file 1: Flow chart of identification strategy
This flow chart illustrates the method used to identify potential examples of gene pairs that have undergone concerted evolution in at least two exons and their intervening intron since at least the human-mouse divergence. The flow chart also outlines the filtering steps utilized to create a potential concerted evolution dataset that was then manually curated to identify the three gene pair examples presented.
