# Supplementary material for: Identifying concerted evolution and gene conversion in mammalian gene pairs lasting over 100 million years
Source: BMC Evol Biol. 2009 Jul 7;9:156. doi: 10.1186/1471-2148-9-156 (PMC2720389; doi:10.1186/1471-2148-9-156)
Supplement: Additional file 2 — Maximum likelihood trees of gene pairs. This file shows maximum likelihood trees of gene pairs created in PAUP. [file 1471-2148-9-156-S2.pdf]

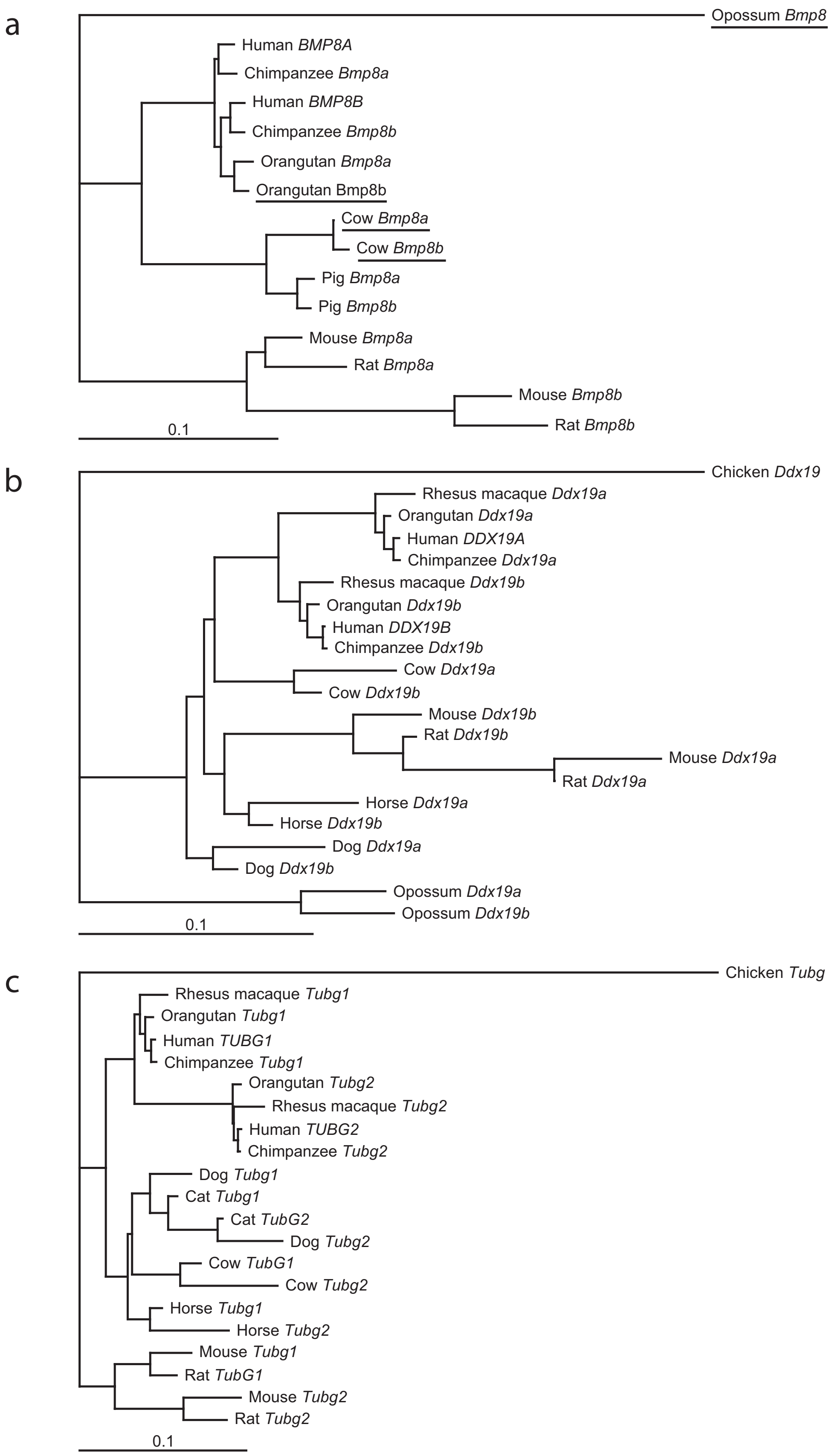

Additional file 2: Maximum likelihood trees of gene pairs

Maximum likelihood trees of gene pairs a) *BMP8A/B*, b) *DDX19A/B* and c) *TUBG1/2* were created in PAUP using the likelihood settings from the best-fit model selected by hLRT in MODELTEST (parameters available upon request). Like the neighbor-joining trees, opossum *Bmp8*, chicken *Ddx19* and chicken *Tubg* were used as outgroups to root the trees. A distance scale is shown below each tree. Underlined species in a) are missing exon 1, preventing the use of this exon in the phylogenetic analysis.
