# Supplementary material for: Identifying concerted evolution and gene conversion in mammalian gene pairs lasting over 100 million years
Source: BMC Evol Biol. 2009 Jul 7;9:156. doi: 10.1186/1471-2148-9-156 (PMC2720389; doi:10.1186/1471-2148-9-156)
Supplement: Additional file 3 — Sliding window analysis calculating percent identity between orthologues and paralogues. This file details a sliding window percent identity analysis that shows the mosaic evolution within the gene pairs. This figure shows the sliding window analysis plot with the percent identities calculated between orthologues and paralogues. [file 1471-2148-9-156-S3.doc]

**Additional file 3: Sliding window analysis calculating percent identity between orthologues and paralogues**

To test our hypothesis of mosaic evolution, we ran a sliding window analysis across the gene pairs. In this analysis, we calculated percent identity within primate (between human and rhesus macaque) and within rodent (between mouse and rat) orthologues and paralogues in sliding windows of 100 bps (with a 5 bps step size) [see Additional file 4]. Averages were calculated for all of the paralogues (blue and green lines in figure) and for all of the orthologues (black and red lines in figure). For *BMP8B*’s macaque orthologue, we were unable to obtain sequence for exon 5 (80 bps), so marmoset sequence was substituted such that a complete gene sequence could be used. From these analyses it is apparent that each sliding window analysis has peaks and valleys in the paralogy plots. For some stretches of windows, the average sequence identity between orthologues (black and red lines) is greater than the average sequence identity between paralogues (blue and green lines). This would be expected for divergent evolution. However, in other stretches of windows, average paralogue identity peaks are greater than average orthologue peaks. These peaks often have a significant number of consecutive windows with 100% identity between paralogues. The high identity of paralogues relative to orthologues is a hallmark of concerted evolution. Hence, much like the phylogenetic trees, these sliding windows show evidence for a mosaic pattern of evolution within the gene pairs, with some exons evolving divergently (where average sequence identity of orthologues > average sequence identity of paralogues) and others evolving concertedly (where average sequence identity of paralogues > average sequence identity of orthologues).
