# Supplementary material for: Identifying concerted evolution and gene conversion in mammalian gene pairs lasting over 100 million years
Source: BMC Evol Biol. 2009 Jul 7;9:156. doi: 10.1186/1471-2148-9-156 (PMC2720389; doi:10.1186/1471-2148-9-156)
Supplement: Additional file 4 — Sliding window of percent identities figure. This figure shows the sliding window analysis plot with the percent identities calculated between orthologues and paralogues [file 1471-2148-9-156-S4.pdf]

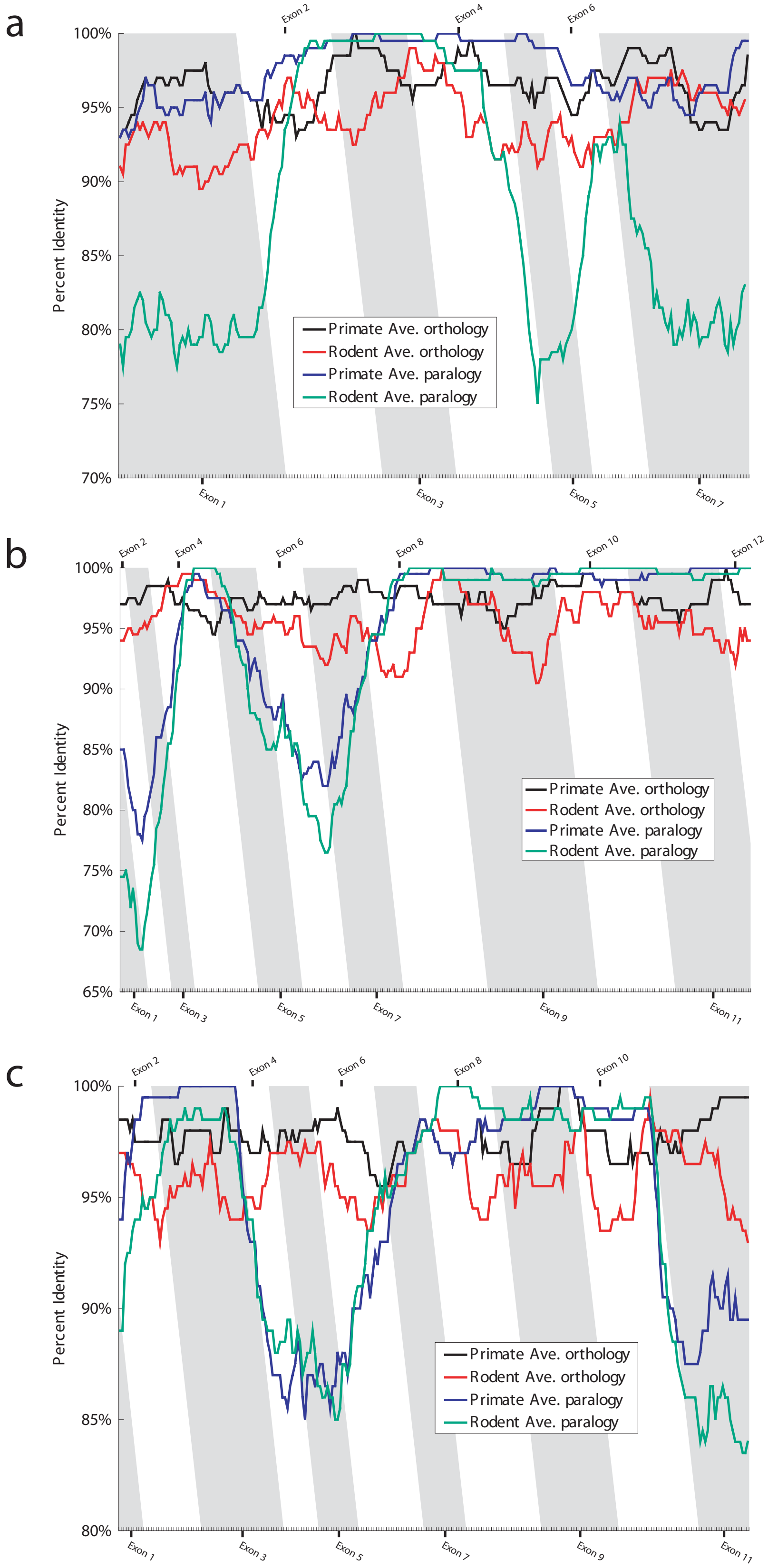

Additional file 4: Sliding window analysis calculating percent identity between orthologues and paralogues
Sliding windows of 100 bps with a 5 bp step size were used to calculate percent identity between gene pairs in a) *BMP8A/B*, b) *DDX19A/B* and c) *TUBG1/2*. Primate comparisons were calculated using human-macaque pairs, while rodent comparisons were calculated using mouse-rat pairs. Average values were calculated from the two gene pair comparisons for both orthologues and paralogues (eg: human *BMP8A* vs human *BMP8B* and macaque *Bmp8a* vs macaque *Bmp8b* were averaged for primate orthology, while human *BMP8A* vs macaque *Bmp8a* and human *BMP8B* vs macaque *Bmp8b* were averaged for primate paralogy). Exon structure is highlighted by alternating grey and white bands behind the plot. Note that marmoset sequence (80 bps) was used to fill in missing exon 5 from the rhesus macaque *Bmp8b* sequence.
