# Supplementary material for: Identifying concerted evolution and gene conversion in mammalian gene pairs lasting over 100 million years
Source: BMC Evol Biol. 2009 Jul 7;9:156. doi: 10.1186/1471-2148-9-156 (PMC2720389; doi:10.1186/1471-2148-9-156)
Supplement: Additional file 5 — Maximum likelihood trees showing mosaic evolution within gene pairs. This file shows maximum likelihood maximum likelihood trees created in PAUP of gene pairs, in which the exons are divided into two categories showing divergent and concerted evolution. [file 1471-2148-9-156-S5.pdf]

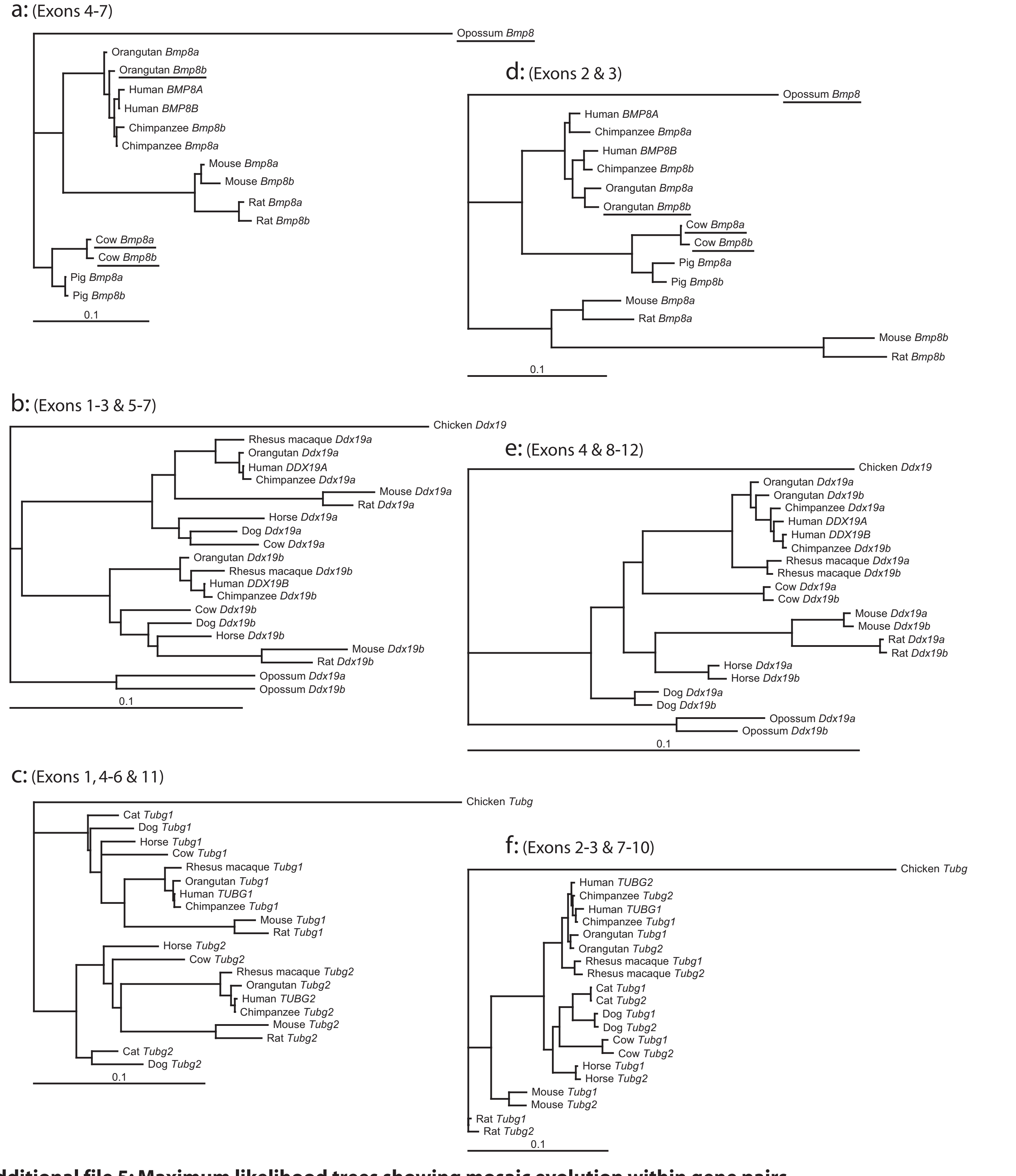

Additional file 5: Maximum likelihood trees showing mosaic evolution within gene pairs

Maximum likelihood trees of gene pairs a) and d) *BMP8A/B*, b) and e) *DDX19A/B* and c) and f) *TUBG1/2* were created in PAUP using the likelihood settings from the best-fit model selected by hLRT in MODELTEST (parameters available upon request). Exons were divided into two categories and phylogenetic trees, showing a), b) and c) divergent evolution or d), e) and f) concerted evolution, are illustrated. Like the neighbor-joining trees, opossum *Bmp8*, chicken *Ddx19* and chicken *Tubg* were used as outgroups to root the trees. A distance scale is shown below each tree. Underlined species in a) and d) are missing exon 1, preventing the use of this exon in the phylogenetic analysis.
