# Supplementary material for: Identifying concerted evolution and gene conversion in mammalian gene pairs lasting over 100 million years
Source: BMC Evol Biol. 2009 Jul 7;9:156. doi: 10.1186/1471-2148-9-156 (PMC2720389; doi:10.1186/1471-2148-9-156)
Supplement: Additional file 6 — Description and percent similarity between gene pairs. This file contains a table containing information about the gene pairs, including their ORF size, number of exons, and the percent identities of regions evolving divergently or concertedly. [file 1471-2148-9-156-S6.doc]

**Additional file 6**: Description and percent similarity between gene pairs

| Gene | # of Exons | Transcript Size (ORF) | # of exons within concerted | Similarity concerted | Similarity divergent |
| --- | --- | --- | --- | --- | --- |
| *BMP8A* | 7 | 1209 | 7 | 98% (1187/1209) | N/A |
| *BMP8B* | 7 | 1209 |
| *DDX19A* | 12 | 1437 | 6 | 99% (962/969) | 86% (407/471) |
| *DDX19B* | 12 | 1440 |
| *TUBG1* | 11 | 1356 | 6 | 98% (824/833) | 87% (460/523) |
| *TUBG2* | 11 | 1356 |
