# Supplementary material for: Identifying concerted evolution and gene conversion in mammalian gene pairs lasting over 100 million years
Source: BMC Evol Biol. 2009 Jul 7;9:156. doi: 10.1186/1471-2148-9-156 (PMC2720389; doi:10.1186/1471-2148-9-156)
Supplement: Additional file 7 — Sliding window analysis calculating dN/dS between paralogues and orthologues. This file shows sliding windows used to calculate dN/dS between gene pairs. [file 1471-2148-9-156-S7.doc]

**Additional file 7: dN/dS analysis using sliding window and PAML approaches**

To examine the dN/dS values in the gene pairs, we conducted a sliding window analysis between primate (human vs macaque, except in *BMP8A/B* where orangutan was used) and rodent (mouse vs rat) orthologues. The analysis was conducted using K-estimator [60] with a window size of 99 bps (33 codons) and a step size of 6 bps (2 codons). The values were then plotted to obtain a graph of dN/dS values across the gene [see Additional file 8]. Interestingly, in *BMP8A*, *BMP8B* and *DDX19B*, peaks with values above one, which could suggest positive selection, are seen in these plots. For *BMP8A*, two peaks above one are seen in the primate analysis, along with a large peak in the rodent. However, only a single peak is seen for *BMP8A* (primate) and *DDX19B* (rodent). As these peaks could indicate regions where positive selection has occurred, we conducted additional analyses using PAML [61]. However, although the results from some analyses show suggestions of positive selection, no significant evidence was found (data not shown). Therefore, there does not appear to be an overlap between the regions undergoing concerted evolution and positive selection of amino acids in these gene pairs.

**References**

60. Comeron JM: **K-Estimator: calculation of the number of nucleotide substitutions per site and the confidence intervals**. *Bioinformatics* 1999, **15**(9):763-764.

61. Yang Z: **PAML: a program package for phylogenetic analysis by maximum likelihood**. *Comput Appl Biosci* 1997, **13**(5):555-556.
