# Supplementary material for: Identifying concerted evolution and gene conversion in mammalian gene pairs lasting over 100 million years
Source: BMC Evol Biol. 2009 Jul 7;9:156. doi: 10.1186/1471-2148-9-156 (PMC2720389; doi:10.1186/1471-2148-9-156)
Supplement: Additional file 8 — Sliding windows calculating dN/dS figure. This figure shows the sliding window analysis plot with the dN/dS values calculated between gene pairs. [file 1471-2148-9-156-S8.pdf]

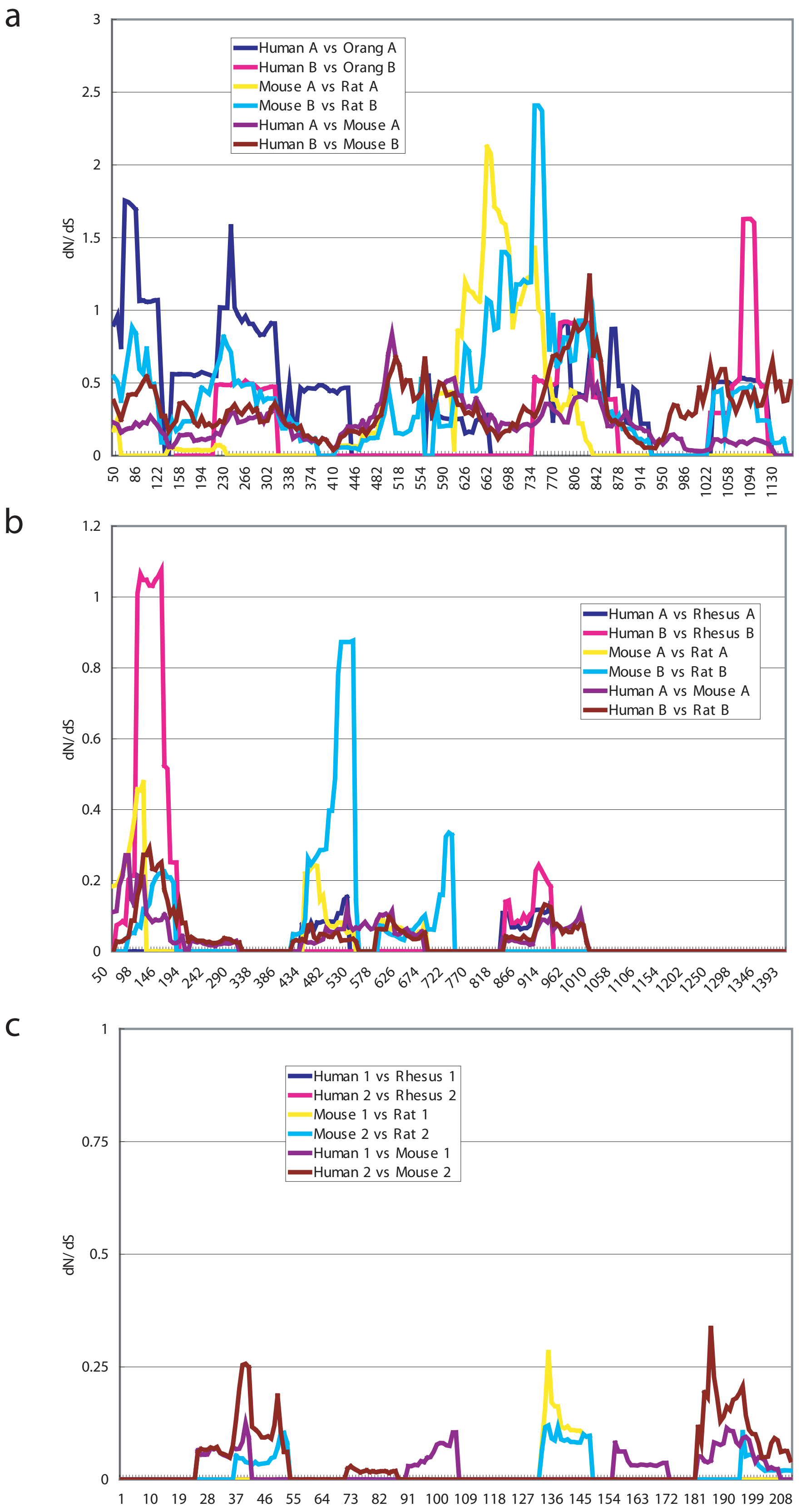

Additional file 8: Sliding window analysis calculating dN/dS between paralogues and orthologues
Sliding windows of 99 bps with a 6 bp step size were used to calculate dN/dS between gene pairs in a) *BMP8A/B*, b) *DDX19A/B* and c) *TUBG1/2*. Values of dN/dS were calculated within the windows using the program K-estimator and plotted across the gene pairs. Primate comparisons were calculated using human-macaque pairs (except in *BMP8A/B* where orangutan was used), while rodent comparisons were calculated using mouse-rat pairs. Although we conducted additional analyses using PAML, no significant evidence of positive selection was found.
