# Supplementary material for: Identifying concerted evolution and gene conversion in mammalian gene pairs lasting over 100 million years
Source: BMC Evol Biol. 2009 Jul 7;9:156. doi: 10.1186/1471-2148-9-156 (PMC2720389; doi:10.1186/1471-2148-9-156)
Supplement: Additional file 9 — Analysis of functional domains. This file contains a description of the analysis looking for overlap between functional domains and regions evolving in concert. [file 1471-2148-9-156-S9.doc]

**Additional file 9: Analysis of functional domains**

We performed an analysis to see if the regions undergoing concerted evolution contained or overlapped with specific functional domains within these genes. To look for overlap between the regions of concerted evolution and functional domains, we used SMART [62-64] and Pfam [65, 66]. These programs identified regions of the protein that contain known domains, which could then be compared to the regions of the protein that have been affected by gene conversion.

For *BMP8A/B*, the two programs predict a signal peptide (within exon 1; a.a.’s 1-24), a TGFβ propeptide (exons 1-3; a.a.’s 27-252), and a TGFβ domain (exons 5-7; a.a.’s 301-402). From Figure 1 and Table 1, we can see that although all seven exons in these genes show some signal of concerted evolution, the most significant values are in exons outside of the TGFβ domain. This is especially true in rodent, where only exons 2-4 shows concerted evolution. This could indicate that there is another functional domain in these exons that has not been previously described.

For *DDX19A/B*, the two programs predict DEXDc (exons 5-9; a.a.’s 110-309) and HELICc (exons 10-11; a.a.’s 346-433) domains. From Figure 1 and Table 1, there doesn’t appear to be a significant overlap with the regions undergoing concerted evolution. Although part of the DEXDc domain and the entire HELICc domain overlap the region of concerted evolution, there is no clear relationship that could indicate the selection for gene conversion limited to a particular domain.

A similar case is seen in *TUBG1/2*, where the two programs predict a Tubulin domain (exons 3-7; a.a.’s 48-247) and a Tubulin_C domain (exons 8-10; a.a.’s 249-393). Our previous analyses indicate that gene conversion is taking place in exons 2-3 and exons 7-10. Hence, again, there is no clear overlap with either of the two domains, although there is some suggestion that the Tubulin domain has avoided concerted evolution while the Tubulin_C domain has continued to undergo gene conversion and has remained homogenized. Again, like *BMP8A/B*, there is a significant signal for gene conversion outside of any of the known domains (exons 2-3) that could contain an uncharacterized domain.

**References**
