# Supplementary material for: Identifying concerted evolution and gene conversion in mammalian gene pairs lasting over 100 million years
Source: BMC Evol Biol. 2009 Jul 7;9:156. doi: 10.1186/1471-2148-9-156 (PMC2720389; doi:10.1186/1471-2148-9-156)
Supplement: Additional file 10 — Analysis of upstream regulatory regions. This file describes the analysis of predicted regulatory regions in the 10 kb of sequence upstream from each gene. It also contains a table showing the predicted regulatory regions in these sequences. [file 1471-2148-9-156-S10.doc]

**Additional file 10: Analysis of upstream regulatory regions**

We performed additional analysis looking for regulatory regions upstream of each gene to try to assess whether gene pairs share promoters or enhancers. We used the UCSC genome browser [51] and examined 10 kb of upstream sequence for each gene using several of the Regulation tracks including FirstEF, Vista Enhancers and TFBS Conserved (Transcription Factor Binding Sites). We also performed mVISTA [52, 53] analysis looking for duplicated or conserved homologous sequences shared between each gene pair in their upstream sequences. From this analysis, it does not appear that many upstream regulatory regions have been conserved between the gene pairs. For *BMP8A/B*, there is a small region of homology shared between the gene pair in the first ~1.5 kb upstream of the genes. Within this region, there is a promoter predicted by FirstEF that appears to be conserved between both genes. Further upstream, there does not appear to be any conserved sequences, enhancers, or transcription factor binding sites. No Vista Enhancers are seen upstream of either gene, while the conserved TFBS sites differ between the two genes. For *DDX19A/B* and *TUBG1/2*, there does not appear to be any conserved homologous sequences in their upstream sequences. Similarly, no VISTA enhancer or TFBS conserved regions are shared between the gene pairs. Thus, apart from one promoter region shared between *BMP8A* and *BMP8B*, it appears that the upstream regulatory regions have diversified throughout the gene pair evolution, even though regions within the genes have evolved in concert.

| **Gene** | **Vista Enhancer** | **TFBS Conserved** |
| --- | --- | --- |
| *BMP8A* | None | RREB1, CDP, GRE, PAX6, HAND1E47 |
| *BMP8B* | None | None |
| *DDX19A* | None | CREB, CREBP1CJUN, CREBP1 |
| *DDX19B* | None | ATF, AHR, YY1, CMYB, SREBP1, USF, ARNT, CHOP |
| *TUBG1* | None | NKX61 |
| *TUBG2* | None | SREBP1 |

**Additional Data File 10 Table: Predicted regulatory regions from UCSC browser in 10 kb of sequence upstream of human genes**

**References**
